# P-1650. Clinical Outcomes among Patients Prescribed Suboptimal Antibiotic Therapy on Discharge to Nursing Homes

**DOI:** 10.1093/ofid/ofae631.1816

**Published:** 2025-01-29

**Authors:** Emily K Short, Brie Noble, Christopher J Crnich, Dominic Chan, Andrea Hildebrand, Thuan Nguyen, Michelle H Zhou, Evita T Santos, YoungYoon Ham, Emily Shephard, Jessina C McGregor, Sally Jolles, Caitlin M McCracken, David T Bearden, Seiko Izumi, Jon P Furuno

**Affiliations:** Oregon State University College of Pharmacy, Portland, Oregon; Mayo Clinic, Scottsdale, Arizona; University of Wisconsin School of Medicine and Public Health, Madison, WI; Legacy Health, Portland, OR; Oregon Health and Science University, Portland, Oregon; Oregon Health and Science University-Portland State University School of Public Health, Portland, Oregon; Oregon State University College of Pharmacy, Portland, Oregon; Yale New Haven Hospital, New Haven, Connecticut; Oregon Health & Science University, Portland, Oregon; Legacy Health, Portland, OR; Oregon State University, Portland, Oregon; University of Wisconsin School of Medicine and Public Health, Madison, WI; Oregon State University College of Pharmacy, Portland, Oregon; Oregon State University/Oregon Health & Sciences University, Portland, OR; Oregon Health and Science University School of Nursing, Portland, Oregon; Oregon State University, Portland, Oregon

## Abstract

**Background:**

Little is known regarding outcomes for patients prescribed antibiotics on discharge from the hospital to nursing homes (NHs). We quantified the association between suboptimal antibiotic prescribing and clinical outcomes at discharge to NHs.

**Methods:**

This was a multicenter, retrospective cohort study of adult (age >18 years) patients prescribed systemic antibiotics on discharge from 9 acute care hospitals in Oregon, Washington, and Wisconsin to a NH from 2016 – 2018. Study data were electronically extracted from hospital and NH electronic health records and supplemented with chart review. Suboptimal antibiotic therapy was determined by an infectious disease pharmacist or physician using clinical information (e.g., antibiotic regimen, indication, and culture results) available at discharge. Outcomes included emergency department (ED) visits, hospital readmissions, or Clostridioides difficile infection (CDI) within 30 days of hospital discharge. Comparison of outcomes between patients with suboptimal and non-suboptimal antibiotic therapy were tested by chi-squared tests.

**Results:**

Among 280 included patients, mean (SD) age was 76.0 (11.5) years, 35.7% (n=100) were male, and median (IQR) inpatient length of stay was 6 (4-9) days. The most prevalent antibiotic indications were urinary tract infections (n =109, 38.9%), lung indications (n=60, 21.4%), and prophylaxis (n=29, 10.4%). Among 317 antibiotic prescriptions, the most frequently prescribed antibiotic classes were cephalosporins (n=83, 29.6%), fluoroquinolones (n=57, 20.4%), and beta-lactam/beta-lactamase-inhibitors (n=40, 14.3%). Fifty-eight patients (20.7%) were prescribed suboptimal antibiotic therapy on hospital discharge. There were no significant differences in the frequency of 30-day ED visits (14% vs 11%, p=0.76) or hospital readmission (19% vs 18%, p=0.94) between patients prescribed and not prescribed suboptimal antibiotics; however, suboptimal antibiotics were significantly associated with CDI within 30 days of discharge (10% vs 2%, p=0.01).

**Conclusion:**

Suboptimal antibiotic prescribing on discharge to NHs was associated with increased risk of CDI within 30 days of discharge. This finding may inform interventions to improve antibiotic prescribing on discharge to NHs.

**Disclosures:**

**YoungYoon Ham, PharmD**, Gilead: Honoraria **Jon P. Furuno, PhD**, Merck & Co., Inc: Grant/Research Support

